# A window into the brain: An in vivo study of the retina in schizophrenia using optical coherence tomography

**DOI:** 10.1016/j.pscychresns.2011.08.011

**Published:** 2012-07-30

**Authors:** Elvina May-Yin Chu, Madhan Kolappan, Thomas R.E. Barnes, Eileen M. Joyce, Maria A. Ron

**Affiliations:** a Department of Neuropsychiatry, University College London, Institute of Neurology, London, UK; b Department of Psychological Medicine, Imperial College, London, UK

**Keywords:** Biological markers, Macular volume, Retinal nerve fibre layer

## Abstract

Retinal nerve fibre layer (RNFL) thickness and macular volume (MV) can be measured in vivo using optical coherence tomography (OCT) providing a “window into the brain”. RNFL and MV are promising biomarkers in neurological diseases. This study explores the potential of RNFL and MV to detect axonal abnormalities in vivo in schizophrenia and their correlations with clinical features. OCT was performed in 49 patients (38 schizophrenia, 11 schizoaffective disorder) and 40 healthy controls matched for age and gender. Group comparisons were made between whole retina and quadrant RNFL thickness and MV using multi-level analyses. In patients, associations were sought between RNFL and MV with symptom severity (positive/negative). Patients and controls had similar whole retina RNFL thickness (*p* = 0.86) and MV (*p* = 0.64), but RNFL in the right nasal quadrant of the schizoaffective group was thinner than in the schizophrenia group (*p* = 0.02). In patients, positive symptom severity was associated with smaller MV (right β = − 0.54, p = 0.02; left β = − 0.49, *p* = 0.04). Normal MV and RNFL thickness suggests unmyelinated axons in patients with schizophrenia/schizoaffective disorder remain unaffected. Longitudinal studies using higher resolution OCT will clarify whether progressive RNFL and MV changes occur and whether they can be used as state or trait markers in schizophrenia.

## 1 Introduction

Schizophrenia lacks a clearly defined or diagnostic neuropathological signature. The best validated neuropathological abnormalities include possible reductions of neuronal density in the thalamus, but not in the cerebral cortex or hippocampus, although in these structures pyramidal neurons may have smaller bodies with reduced dendritic arborisation and spines (Harrison and Weinberger, 2005). Reductions in the number of some interneurons and in the number and function of oligodendrocytes, relevant to myelination and neuronal and synaptic integrity, complete the picture (Harrison and Weinberger, 2005). In keeping with these findings, neuroimaging studies have reported grey matter volume deficits in those with chronic schizophrenia (Ellison-Wright and Bullmore, 2009), those in their first episode of psychosis (Bangalore et al., 2009), in a prodromal phase of illness (Pantelis et al., 2003), or at high genetic risk of schizophrenia (Witthaus et al., 2009). Childhood onset schizophrenia also results in progressive grey matter volume loss in the early years of illness (Sporn et al., 2003). Compromised white matter integrity rather than volume loss has been reported in first episode (Price et al., 2007) and chronic schizophrenia (Kanaan et al., 2009).

Longitudinal imaging studies have also suggested that at least in some patients there may be an accelerated loss of grey matter in comparison with healthy controls immediately after the first episode of schizophrenia (Sun et al., 2009), with similar results documented in chronic patients (Brans et al., 2008) and in those at ultra high risk, even before the first episode of psychosis (Takahashi et al., 2010). However, the precise neuropathological changes that underlie these neuroimaging findings remain to be determined.

In vivo visualisation of the retinal nerve fibre layer (RNFL) can be achieved by optical coherence tomography (OCT), a non-invasive, fast imaging technique used to monitor retinal changes in glaucoma (Huang et al., 1991). There are no contra-indications to OCT, which opens a “window into the brain” by allowing measurement of RNFL thickness and macular volume (MV). The RNFL is composed of unmyelinated axons that traverse the retina, with the highest axonal density contained in the macula. OCT has recently been applied to the study of neurological conditions with diffuse and progressive brain pathology.

RNFL thinning has been described in patients with mild cognitive impairment without dementia (Paquet et al., 2007). It correlates with the severity of cognitive impairment in patients with Alzheimer's disease (Iseri et al., 2006), and Berisha et al. (2007) have reported it in the early stages of Alzheimer's disease. RFNL thinning is also present in patients with Parkinson's disease, in whom loss of dopaminergic neurons is known to occur, not only in the cortex and basal ganglia but also in the retinal ganglion cells (Altintas et al., 2007). OCT reveals a variable degree of RNFL thinning (mean ~ 20%) following an episode of optic neuritis (Trip et al., 2005). Reduced RNFL thickness is also described in multiple sclerosis (MS), even in patients without a prior history of optic neuritis and this correlates with magnetic resonance imaging (MRI) measures of brain atrophy (Siger et al., 2008). In healthy subjects, RNFL thickness has been reported to be associated with cognitive performance, particularly in those below 40 years of age (van Koolwijk et al., 2009). In glaucoma, around 20% RNFL thinning was reported at 2-month follow-up after onset of the disease (Fang et al., 2007), while rate of RNFL thinning in a longitudinal study of chronic glaucoma was less than 1% a year (Leung et al., 2011).

In schizophrenia, widespread; albeit subtle, neuropathological abnormalities, together with electroretinogram (ERG) changes indicative of reduction in rod photoreceptors or changes in inter-neuronal retinal architecture (Balogh et al., 2007), have also been described in those at high genetic risk of schizophrenia (Hébert et al., 2009) and in autism, (Ritvo et al., 1988). Hence there is good reason for using OCT to search for disease biomarkers.

We present here the first study, to our knowledge, to use OCT to study the retina in patients with schizophrenia and schizoaffective disorder. We hypothesised that retinal changes indicative of schizophrenia-related neuropathology would be present and quantifiable in our patients. We also looked for associations between OCT findings and disease symptoms.

## 2 Methods

### 2.1 Subjects

Patients recruited to this study were selected from a larger cohort (the West London First Episode Psychosis study) and had a firmly established diagnosis of schizophrenia or schizoaffective disorder using the DIP-DM Diagnostic Interview for Psychosis (Jablensky, 2000), which includes items from the Operational Criteria Checklist for Psychosis (OPCRIT) (McGuffin et al., 1991) and the WHO Schedules for Clinical Assessment in Neuropsychiatry (SCAN) (Wing et al., 1990). Substance abuse and dependence were established using the Alcohol and Drug Use Scales, (Drake et al., 1990). Two research nurses conducted the interviews, and patients were clinically reviewed by two experienced consultant psychiatrists (EJ and TB) to confirm the diagnosis 1 year after inclusion in the study.

Forty-nine patients (36 males, 13 females), mean age 29.9 years (S.D. ± 8.74) with diagnoses of schizophrenia (38 patients) or schizoaffective disorder (11 patients) and a mean illness duration of 4.4 years (S.D. ± 3.6) at the time of OCT scanning were recruited. Forty patients were prescribed antipsychotics (39 atypical and 1 typical), 10 patients were prescribed antidepressants and four mood-stabilising medication concurrent with an antipsychotic, two were prescribed mood stabilisers only, one was prescribed night sedation only, and five were unmedicated. Scores on the Schedules for the Assessment of Positive and Negative Symptoms (SAPS and SANS) (Andreasen, 1981; Andreasen, 1983) were available for a subset of 32 patients. Severity of negative symptoms ranged from 0 to 16, mean 3.7, and positive symptoms ranged from 0 to 10, mean 2.

Forty healthy comparison subjects, matched for age (mean 29.5 years, S.D. ± 6.12) and gender (25 males, 15 females), were recruited.

All subjects included in the study had a normal ophthalmic examination including Humphrey 30–2 threshold visual fields test and logMAR visual acuity test. There were no differences between groups in visual fields: right (*t* = − 0.45, d.f. = 73, *p* = 0.66), left (*t* = − 0.58, d.f. = 73, *p* = 0.56) or visual acuity: right (*t* = 1.77, d.f. = 76, *p* = 0.08), left (*t* = 1.76, d.f. = 76, *p* = 0.08). In patients the mean visual fields (S.D.) were right = − 1.11 (1.35), lef t= − 1.15 (1.33) and in controls right = − 1.06 (1.45), left = − 1.11 (1.35). In patients the mean visual acuities (S.D.) were right = 0.004 (0.11), left = 0.004 (0.12) in controls right = − 0.029 (0.10) and left = − 0.029 (0.11). Intraocular pressures were not recorded, but there was no evidence of optic disc cupping in any of our subjects.

#### 2.1.1 Exclusion criteria

Exclusion criteria for all subjects were as follows: a) concurrent or previous systemic disease (e.g. diabetes or autoimmune disease) that could involve the eyes, b) a history of neurological or ophthalmological disease known to affect the visual pathway (e.g. glaucoma), c) high myopia (<−6D) as this may cause artefactual reduction in RNFL thickness and difficulty in fixation, d) previous head injury with loss of consciousness, and e) drug or alcohol dependence. Ethical approval was granted by the Joint Ethics committee of the UCL Institute of Neurology and University College Hospital NHS Foundation Trust, and this study was carried out in accordance with The Code of Ethics of the World Medical Association (Declaration of Helsinki).

### 2.2 Optical coherence tomography

Using OCT, a cross-sectional image of the retina is produced by measuring the echo time delay of back-scattered infra-red light after it has passed into the eye and is bounced back using a low coherence light source and interferometer. OCT was performed by a neurologist (MK) trained and experienced in using the Stratus OCT3 device and software (Carl Zeiss Meditec Inc., California, USA) at UCL Institute of Neurology. RNFL images were acquired for each eye by taking a circumpapillary scan of 3.4-mm diameter to effectively intercept all nerve fibres converging toward the optic disc while avoiding inaccurate measurements resulting from peripapillary atrophy (Schuman et al., 1996).

#### 2.2.1 Retinal nerve fibre layer and macular volume measures

**Fig. 1:**
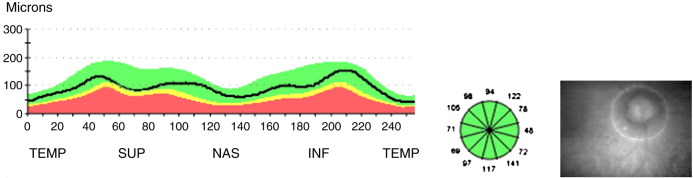
Example of RNFL measurements taken from a healthy eye. Line of graph shows RNFL thickness of the scanning circle as seen around the optic nerve head in the photograph on the right. *X* axis = position on scanning circle and *Y* axis = RNFL thickness at different positions. TEMP 169 = temporal, SUP = superior, NAS = nasal, INF = inferior. The colours represent normal distribution percentiles. Green = 95 to 5%, yellow = 5 to 1%, red = 1 to 0%.

The thickness of the RNFL quadrants (temporal, superior, nasal and inferior), were calculated by the OCT device software and represented by a line graph indicating RNFL thickness at all sections of the scanning circle (Fig. 1). With the Fast RNFL protocol, the mean of three circular 3.4-mm diameter scans, centred on the optic disc, was used to express RNFL thickness. MV was measured in each eye by taking six consecutive radial linear scans centred on the fovea, to provide six sets of equally spaced and intersecting scans, and to obtain a single average value (using the fast macular thickness map scanning protocol). The Stratus OCT3 device assigns a signal strength (10 being the maximum) to OCT images. These were rejected if signal strength was less than 7 or when the difference in signal strength between images for the two eyes was greater than 2 to ensure consistency in the quality of scans obtained.

### 2.3 Statistical analysis

Independent samples *t*-tests and chi-squared tests were used to compare age and gender between all patients and controls.

A generalised linear mixed model (GLMM) approach was used to compare whole retina RNFL thickness, quadrant RNFL thickness and MV between three groups (controls, schizophrenia patients and schizoaffective disorder patients). Multilevel models were used to account for the fact that observations within each patient (i.e. right and left eyes) were not independent, while also controlling for possible confounding effects of age, gender and disease duration (Laird and Ware, 1982). Where association was shown between eye and disease subtype, linear regression analysis was used to investigate the effect of disease subtype on each eye independently.

In the patient group, multiple linear regression analysis was used to investigate the relationship between severity of positive and negative symptoms with measures of RNFL thickness and MV in both eyes, while controlling for age and gender. Statistical significance was reported at *p* < 0.05.

### 2.4 Power calculation

Power calculation was based on a study reporting subtle decreases in RFNL thickness in the unaffected eyes of patients with MS due to subclinical axonal damage (Fisher et al., 2006). We estimated that in order to detect a mean between-group difference in RNFL thickness of 9 μm with a standard deviation of ± 14 μm and a standardised difference of 0.64, a sample size of 38 patients and 38 controls would be needed to achieve an 80% probability of detecting differences between the two groups at a 5% significance level. Based on Henderson et al. (2008), with a mean difference in MV of 0.27 mm³ between groups, our sample size would give a 70% probability of detecting a group difference at the 5% level of significance.

## 3 Results

Patients and controls were matched for age (*t* = 0.27, d.f. = 85, *p* = 0.79) and gender (χ² = 0.27, *p* = 0.36). Mean and 95% confidence intervals for whole retina RNFL, quadrant RNFL and MV measures in controls, schizophrenia and schizoaffective disorder groups are reported in Table 1.

### 3.1 Group comparisons

Using multi-level analyses, patients with schizophrenia or schizoaffective disorder did not differ from controls in whole retina RNFL or MV measurements (Table 2). The left nasal quadrant RNFL appeared thinner than the right across all groups and also showed evidence of an interaction between eye and disease subtype (*p* = 0.002), with significant difference in right but not left nasal quadrant RNFL thickness between the patient groups. After adjustment, schizoaffective disorder patients had a thinner right nasal quadrant (mean − 17.9 μm, 95% CI = − 31.0 to − 4.9) than those with schizophrenia (*p* = 0.02). Left temporal quadrant RNFL appeared thinner than the right across all groups, and there was an interaction between side and disease subtype (*p* = 0.009), but there was no difference in right (*p* = 0.18) or left (*p* = 0.23) temporal quadrant RNFL thickness between groups.

### 3.2 Symptom severity

In patients, multiple linear regression showed a moderate association between lower MV and positive symptom severity (right β = − 0.54, *p* = 0.02; left β = − 0.49, *p* = 0.04). When only those patients with schizophrenia were included in the analysis, a strong association was seen (right β = − 0.85, *p* = 0.04; left β = − 0.85, *p* = 0.05). MV was not associated with severity of negative symptoms, and RNFL was not associated with positive or negative symptom severity.

## 4 Discussion

This study did not detect any differences in whole retina RNFL thickness or MV between patients with schizophrenia or schizoaffective disorder and healthy controls. These negative findings suggest that in patients with schizophrenia or schizoaffective disorder there is no detectable loss of unmyelinated axons in the retina in the early years after disease onset. Based on average values from both eyes for mean and standard deviations of the RNFL thickness of our subjects, a retrospective sample size calculation showed that at least 300 subjects would have been required in each arm of the study to detect any significant differences between groups, had they been present, with 80% power at a 5% level of significance. We therefore conclude that using this OCT methodology, RNFL variations are too subtle to be of value as a biological marker for schizophrenia.

Differences in RNFL thickness were seen in the right nasal quadrant between patients with schizophrenia and schizoaffective disorder. Differences in temporal quadrant RNFL, however, were not associated with disease subtypes. These unexpected results cannot be explained by age or gender differences and should be interpreted with caution given the small number of patients with schizoaffective disorder in this study. Possible medication effects cannot be excluded, as half of the schizoaffective patients were also prescribed antidepressants or mood stabilisers as adjuncts to atypical antipsychotics.

The association between severity of positive symptoms and MV in patients with schizophrenia needs replicating in a larger sample as it may represent state-dependent abnormalities similar to those reported by an ERG study (Balogh et al., 2007) that described transient retinal changes (i.e. decreased amplitude of the a-wave indicative of altered early visual information processing) that which during acute relapses correlated with the severity of positive symptoms and were attributed to state-dependent alterations in phospholipid metabolism and/or impaired dopaminergic transmission.

Our results also suggest that axonal damage may be less important than myelin abnormalities in explaining the frequently reported reductions in brain volume in schizophrenia. There is growing evidence for disruption of white matter integrity in the early stages of schizophrenia (Price et al., 2006, 2007; Pérez-Iglesias et al., 2010), which lends support to this possibility. The negative findings are also in keeping with our earlier studies in patient samples overlapping the one described here, which failed to demonstrate differences in cortical volume between patients and controls using surface-based morphometry (Gutiérrez-Galve et al., 2010) and only fronto-temporal white matter volume loss with no cortical changes observed when using magnetization transfer imaging or volumetric MRI (Price et al., 2006).

The main limitation of this study is low OCT resolution, which may be inadequate to detect the subtle abnormalities that may be present in the early years of schizophrenia. Transverse resolution can reach a maximum of 10 μm in tissue and 15 μm in air using the Stratus OCT device (Fujimoto et al., 2004); hence, sensitivity is too low to visualise individual axons, which are typically 1 μm in diameter. This point has been made by Naismith et al. (2009), who detected RNFL thinning in optic neuritis in only 60% of cases, and detection rates were lower in those with good recovery. Newer technology using spectral domain OCT is faster and offers better axial resolution (5 μm compared to 10 μm) and more accurate macular thickness measurement than the Stratus OCT3 device used in our study.

Most of our patients were receiving atypical antipsychotic medication at the time of data collection, and it is impossible to exclude the potential neuroprotective effects of these drugs (Navari and Dazzan, 2009), which may have obscured minor differences in RNFL between the groups.

Despite mainly negative findings from this first exploratory study, there is a need for further OCT investigations to examine possible RNFL thinning and decreased MV in schizophrenia patients with longer illness duration. A longitudinal study would additionally be able to detect whether progressive retinal changes occur, at which stage of the disease this may happen and whether these retinal measures could be utilised as a trait or state marker of schizophrenia or schizoaffective disorder.

## Figures and Tables

**Table 1:** Whole retina RNFL, quadrant RNFL and MV measures within groups.

OCT measures	Controls	Schizophrenia	Schizoaffective
	Mean (95% CI)	Mean (95% CI)	Mean (95% CI)
Whole retina RNFL (μm)			
Right eye	100.71 (97.49–103.93)	100.90 (97.10–104.70)	97.19 (89.23–105.15)
Left eye	101.24 (97.55–104.94)	99.11 (95.26–102.95)	97.72 (89.66–105.78)

Temporal quadrant RFNL (μm)			
Right eye	70.63 (66.45–74.80)	66.08 (62.24–69.91)	70.18 (61.41–78.95)
Left eye	64.50 (61.44–67.56)	67.82 (64.43–71.20)	64.45 (57.01–71.90)

Superior quadrant RNFL (μm)			
Right eye	128.03 (123.20–132.85)	125.82 (119.84–131.79)	123.00 (108.00–138.00)
Left eye	127.70 (123.22–132.18)	127.74 (122.26–133.21)	127.45 (111.94–142.97)

Nasal quadrant RNFL (μm)			
Right eye	81.53 (76.02–87.03)	88.53 (82.58–94.47)	70.45 (60.96–79.95)
Left eye	83.17 (77.12–89.23)	76.32 (69.76–82.87)	77.55 (69.07–86.02)

Inferior quadrant RNFL (μm)			
Right eye	121.53 (116.56–126.49)	124.13 (118.23–130.03)	124.91 (115.06–134.76)
Left eye	124.28 (119.35–129.20)	123.74 (116.80–130.67)	121.55 (112.29–130.80)

Macular volume (mm³)			
Right eye	6.81 (6.71–6.92)	6.85 (6.72–6.97)	6.90 (6.65–7.16)
Left eye	6.91 (6.81–7.02)	6.91 (6.78–7.03)	6.96 (6.67–7.24)

**Table 2:** Multi-level analyses of whole retina RNFL, quadrant RNFL and MV.

	Unadjusted analysis		Adjusted analysis	
Whole retina RNFL	Regression coefficient (95% CI)	*p*-value	Regression coefficient (95% CI)	*p*-value
Disease type:		0.63		0.86
Control	Ref		Ref	
Schizophrenia	− 0.97 (− 5.79, 3.85)		− 0.78 (− 5.58, 4.03)	
Schizoaffective disorder	− 3.52 (− 10.76, 3.73)		− 1.93 (− 9.32, 5.47)	
Eye; left versus right	− 0.46 (− 1.78, 0.86)	0.50	− 0.46 (− 1.78, 0.86)	0.50
Age; for each 1 year older	− 0.38 (− 0.66, − 0.10)	0.008	− 0.38 (− 0.67, − 0.08)	0.01
Gender; male versus female	− 2.72 (− 7.54, 2.10)	0.27	− 3.13 (− 8.13, 1.87)	0.22
Disease duration; for each year longer	− 1.01 (− 1.85, − 0.16)	0.02	–	–
Temporal quadrant RNFL				
Disease type:		0.96		0.79
Control	Ref		Ref	
Schizophrenia	− 0.62 (− 4.91, 3.68)		− 1.33 (− 5.76, 3.10)	
Schizoaffective disorder	− 0.24 (− 6.70, 6.21)		0.68 (− 6.14, 7.50)	
Eye; left versus right^a^	− 2.72 (− 5.28, − 0.16)	0.04	− 2.72 (− 5.28, − 0.16)	0.04
Age; for each 1 year older	− 0.00 (− 0.26, 0.26)	0.99	− 0.01 (− 0.29, 0.26)	0.92
Gender; male versus female	2.66 (− 1.61, 6.93)	0.22	3.19 (− 1.42, 7.80)	0.18
Disease duration; for each year longer	− 0.65 (− 1.37, 0.06)	0.07	–	–
Superior quadrant RNFL				
Disease type:		0.87		0.83
Control	Ref		Ref	
Schizophrenia	− 1.09 (− 8.02, 5.85)		− 2.10 (− 9.22, 5.01)	
Schizoaffective disorder	− 2.64 (− 13.06, 7.79)		0.36 (− 10.58, 11.31)	
Eye; left versus right	1.22 (− 1.48, 3.93)	0.37	1.22 (− 1.48, 3.93)	0.38
Age; for each 1 year older	− 0.34 (− 0.76, 0.07)	0.10	− 0.36 (− 0.81, 0.08)	0.11
Gender; male versus female	2.09 (− 4.86, 9.03)	0.56	2.52 (− 4.89, 9.92)	0.51
Disease duration; for each year longer	− 1.75 (− 3.00, − 0.49)	0.006	–	–
Nasal quadrant RNFL				
Disease type:		0.25		0.52
Control	Ref		Ref	
Schizophrenia	0.07 (− 6.91, 7.05)		− 0.40 (− 7.61, 6.82)	
Schizoaffective disorder	− 8.35 (− 18.84, 2.14)		− 6.39 (− 17.49, 4.72)	
Eye; left versus right^a^	− 3.60 (− 7.56, 0.37)	0.08	− 3.60 (− 7.56, 0.37)	0.08
Age; for each 1 year older	− 0.37 (− 0.79, 0.05)	0.09	− 0.29 (− 0.74, 0.16)	0.21
Gender; male versus female	1.73 (− 5.36, 8.83)	0.63	0.45 (− 7.06, 7.96)	0.91
Disease duration; for each year longer	− 1.19 (− 2.36, − 0.02)	0.05	–	–
Inferior quadrant RNFL				
Disease type:		0.96		0.94
Control	Ref		Ref	
Schizophrenia	1.03 (− 5.98, 8.05)		0.95 (− 6.31, 8.20)	
Schizoaffective disorder	0.33 (− 10.22, 10.87)		1.79 (− 9.37, 12.94)	
Eye; left versus right	0.65 (− 2.20, 3.51)	0.65	0.65 (− 2.20, 3.51)	0.66
Age; for each 1 year older	− 0.26 (− 0.68, 0.16)	0.22	− 0.28 (− 0.73, 0.17)	0.23
Gender; male versus female	− 1.06 (− 8.09, 5.97)	0.77	− 1.24 (− 8.79, 6.30)	0.75
Disease duration; for each year longer	− 0.65 (− 1.95, 0.66)	0.33	–	–
MV				
Disease type:		0.85		0.64
Control	Ref		Ref	
Schizophrenia	0.01 (− 0.14, 0.16)		0.00 (− 0.16, 0.16)	
Schizoaffective disorder	0.07 (− 0.16, 0.30)		0.11 (− 0.13, 0.36)	
Eye; left versus right^b^	0.08 (0.05, 0.11)	< 0.0001	0.08 (0.05, 0.11)	< 0.0001
Age; for each 1 year older	− 0.00 (− 0.01, 0.01)	0.46	− 0.00 (− 0.01, 0.01)	0.34
Gender; male versus female	0.04 (− 0.12, 0.19)	0.63	0.06 (− 0.11, 0.22)	0.51
Disease duration; for each year longer	− 0.04 (− 0.07, − 0.01)	0.005	–	–

a Quadrant RNFL thinner in left than right eye.

b MV smaller in left eye but no evidence for interaction between eye and disease type (*p* = 0.47).
